# Novel roles of RNA-binding proteins in drug resistance of breast cancer: from molecular biology to targeting therapeutics

**DOI:** 10.1038/s41420-023-01352-x

**Published:** 2023-02-09

**Authors:** Yinghuan Cen, Letian Chen, Zihao Liu, Qun Lin, Xiaolin Fang, Herui Yao, Chang Gong

**Affiliations:** 1 Guangdong Provincial Key Laboratory of Malignant Tumor Epigenetics and Gene Regulation, Sun Yat-Sen Memorial Hospital, Sun Yat-Sen University, 510120 Guangzhou, China; 2 Breast Tumor Center, Sun Yat-Sen Memorial Hospital, Sun Yat-Sen University, 510120 Guangzhou, China; 3 Department of Breast and Thyroid Surgery, Shenzhen People’s Hospital, The Second Clinical Medical College of Jinan University, The First Affiliated Hospital of Southern University of Science and Technology, 518020 Shenzhen, China

**Keywords:** Breast cancer, Cancer therapeutic resistance

## Abstract

Therapy resistance remains a huge challenge for current breast cancer treatments. Exploring molecular mechanisms of therapy resistance might provide therapeutic targets for patients with advanced breast cancer and improve their prognosis. RNA-binding proteins (RBPs) play an important role in regulating therapy resistance. Here we summarize the functions of RBPs, highlight their tremendously important roles in regulating therapy sensitivity and resistance and we also reveal current therapeutic approaches reversing abnormal functions of RBPs in breast cancer.

## Facts

- RBPs exert their regulatory roles in several biological processes.
- Dysregulation of RBPs is involved in therapy resistance of breast cancer.
- Therapeutic approaches targeting key RBPs can reverse therapy resistance of breast cancer.

## Open questions

- What are the differences between canonical RBPs and noncanonical RBPs?
- What are the mechanisms of action of RBPs involved in therapy resistance of breast cancer?
- Would the combination of chemotherapy and approaches reversing abnormality of RBPs improve prognosis of breast cancer patients?

## Introduction

Based on the latest global cancer statistics, breast cancer (BC) had become the most common cause of cancer-related deaths among female worldwide [1]. Although novel chemotherapies, targeted therapies and anti-cancer drugs for BC are continually being updated, drug resistance still occurs and is now the main cause of death for BC patients [2, 3]. Up to now, BC can be classified into three subtypes: hormone receptor (HR)-positive subtype, human epidermal growth factor receptor 2 (HER2)- overexpressing subtype and triple-negative subtype based on HR and HER2 status [4]. Drug resistance is a main reason for cancer therapy failure, but the potential mechanisms are not yet fully understood [5]. Endocrine therapy, as a standard treatment for HR-positive patients, has achieved remarkable success, but there are about 20–30% of patients developing endocrine therapy resistance [6]. These endocrine therapy-resistant cancer cells are either intrinsically resistant phenotype or developing acquired resistance after long-time exposure [6]. HER2-amplified subtype accounts for 20% of BC. The combination of chemotherapy and anti-HER2 therapy serves as a standard treatment for HER2-amplified subtype [7]. Around 25% of advanced HER2-overexpressing BC patients present with primary resistance or develop acquired resistance to HER2-directed treatments [8]. HER2-directed treatment resistance will lead to short progress free survival and overall survival [9]. Triple-negative breast cancer (TNBC) is characterized by the absence of HR and HER2. It easily relapses and often progresses to an advanced stage because of therapy resistance [10]. Therefore, it is of great importance to explore the mechanisms of conventional treatment resistance of BC.

Proteins that directly interact with RNA are defined as RNA-binding proteins (RBPs). RBPs can bind with RNAs to form ribonucleoprotein complex to adjust gene expression post-transcriptionally [11, 12]. RBPs are involved in regulating several processes such as RNA alternative splicing, RNA decay, RNA translation, RNA translocation and so on. Over the last decades, researches have shown that dysregulation of RBPs contributes to cancer treatment resistance. RBPs can regulate endocrine therapy resistance, chemotherapy resistance, targeted therapy resistance and immunotherapy resistance by conducting RNA alternative splicing, RNA decay, RNA translation, RNA translocation and other mechanisms [11]. Consequently, exploring mechanisms of drug resistance induced by abnormalities of RBPs may provide a potential approach to improve the prognosis of BC [13].

In this review, we summarize the functions of RBPs, emphasize their regulatory roles in treatment resistance and discuss RBPs as potential targets for BC treatment.

## Characteristics of RNA binding protein

RBP is endowed with the ability to bind with RNA through special sequences within its molecular structure. There are several online databases to classify eukaryotic RBPs and we summarize these resources in Table 1. RBPs can be classified into canonical and noncanonical RBPs depending on the existence of identified RNA-binding domains (RBDs). RBDs are the functional units responsible for RNA binding. By employing different types of RBDs cooperatively or independently, RBPs will enjoy increased binding to different types of RNAs with enhanced RNA binding affinity as well as specificity [14]. Canonical RBPs recognize sequence-specific RNA motifs through their RBDs. Here we present several classical RBDs including RNA recognition motif (RRM), K homology (KH) domain, double-stranded RNA-binding domain (dsRBD) and zinc finger domain.

**Table 1:** Summary of online databases for eukaryotic RBPs.

Name	Website	Species involved	Description
EuRBPDB	(http://eurbpdb.syshospital.org/)	162 species (homo sapiens, mus musculus, fly, worm, yeast, et al.)	A database for eukaryotic RBPs. Include known cancer associated RBPs from public sources.
RBPDB	(http://rbpdb.ccbr.utoronto.ca/)	Homo sapiens, mus musculus, drosophila melanogaster, caenorhabditis elegans	A collection of RBPs linked to a curated database of published observations of RNA binding, categorized by RBP domain or species.
RBPbase	(https://rbpbase.shiny.embl.de)	Homo sapiens, arabidopsis thaliana, caenorhabditis elegans, drosophila melanogaster, mus musculus, senatus concilium	A database that integrates high-throughput RBP detection studies.
RBPmap	(http://rbpmap.technion.ac.il/)	Specifically for homo sapiens, mus musculus and drosophila melanogaster	A Server for prediction and mapping of RBPs binding sites on RNA sequences
CLIPdb	(http://clipdb.ncrnalab.org)	Homo sapiens, mus musculus, zebrafish, fly, worm, arabidopsis, and yeast	A database for the binding sites collection of RBPs and their functional annotations
RBPsuite	(http://www.csbio.sjtu.edu.cn/bioinf/RBPsuite/)	Homo sapiens	A database contains iDeepS and CRIP developed for predicting RBP binding sites on linear RNAs and on circular RNAs respectively.
RBPTD	(www.rbptd.com/#/)	Homo sapiens	A database for the expression levels and prognosis data of cancer-related RBPs among 28 cancers.
oRNAment	(http://rnabiology.ircm.qc.ca/oRNAment/.)	Homo sapiens, caenorhabditis elegans, danio rerio, drosophila melanogaster and mus musculus	A database of putative RBP binding sites instances in both coding and non-coding RNA.
RBP2GO	(https://rbp2go.dkfz.de/)	13 species (homo sapiens, mus musculus, danio rerio, drosophila melanogaster, caenorhabditis elegans, et al.)	A database dedicated to the analysis of RBPs, their interactions and functions.

### RRM

RRM, also known as ribonucleoprotein consensus sequence, is the most abundant and the best characterized RBD [15]. In Protein Data Base, over 500 structures of RRMs are characterized [15]. A typical RRM consists of 80–90 amino acids and folds into a β1α1β2β3α2β4 topology that forms two α-helices against a four-stranded antiparallel β-sheet, where the RNA recognition usually occurs [16]. RRM-containing proteins bind to diverse RNAs by recognizing 2–8 nucleotides of a single-stranded RNA [17]. Each RRM has its own sequence preferences. Fused in sarcoma family proteins are comprised of RRMs, prion-like domains and other RBDs such as zinc finger domain [18]. The combination of RRMs with other domains allows the binding between fused in sarcoma family proteins and a wide range of nucleic acids in a length-dependent manner [19, 20].

### KH domain

KH domain has a conserved GxxG loop which links two α-helices and β-strands. KH domain binds to polypyrimidine or A/C-rich RNAs with high affinity [21]. KH domain typically recognizes 4 nucleotides of RNAs. Besides, a single RBP can have repeated KH domains to increase binding. For example, vigilins are characterized by the presence of 14 or 15 KH domains and vigilins bind to nucleic acids promiscuously, including transfer RNAs, ribosomal RNAs, small nuclear and small nucleolar RNAs, non-coding RNAs, and mitochondrial RNAs [22, 23].

### dsRBD

dsRBD, which consists of ~65–70 amino acids, folds into a α1β1β2β3α2 structure forming an antiparallel β-sheet flanked by α-helices on one face [24]. The central role of dsRBD is to bind to double-stranded RNA (dsRNA) or highly structured RNAs [24]. RBPs with one or more dsRBDs interact with dsRNA by sensing the double helix RNA structures [24]. dsRBD-containing proteins include ribonuclease IIIs [25] and RNA-editing enzyme such as adenosine deaminases acting on RNA [26].

### Zinc finger domain

Zinc finger domain is composed of around 30 amino acids and forms a simple ββα topology [27]. The residues in the β-hairpin and α-helix of ββα structure are coordinated by a Zn^2+^ ion [27]. Zinc finger domains are capable of binding a diverse range of molecules including DNA, RNA, protein and lipid [28]. The arrangements of cysteines (C) and histidines (H) such as CCHC, CCCH, CCCC and CCHH determines RNA-binding preference of zinc finger domains. For example, subtypes with multiple CCCH and CCCC are inclined to bind 3 nucleotides repeats of RNAs while subtypes with abundant CCHH interact with single-stranded RNAs as well as dsRNAs [28, 29].

As for noncanonical RBPs, several studies discovered a number of noncanonical RBPs which are characterized by the lack of established RBDs [30]. It was challenging to discover noncanonical RBPs without bias because of their lack of RBDs. Since limitations have been overcome with the development of system-wide approaches, numerous noncanonical RBPs have been identified [31]. For instance, a E3 ligase Tripartite Motif 25 interacts with RNA and functions as an RNA-specific RBP cofactor to mediate RNA uridylation [32]. These noncanonical RBPs bind with RNAs via intrinsically disordered regions or mononucleotide- or dinucleotide-binding domains with low sequence complexity [33].

## The function of RBPs

**Fig. 1:**
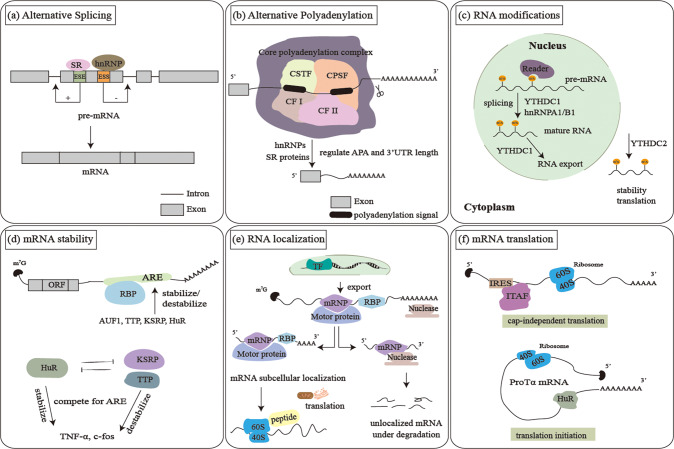
Basic functions of RBPs in the post-transcriptional regulation of gene expression. RBPs can determine the fate of RNAs by controlling various events. Schematic diagrams of the functions of RBPs and the representative ones are shown in **a** alternative splicing, **b** alternative polyadenylation, **c** RNA modifications, **d** mRNA stability, **e** RNA localization, **f** mRNA translation.

In the following, we introduce functions of RBPs and briefly describe the regulatory processes (Fig. 1).

### Pre-mRNAs alternative splicing (AS)

Since coding genes of eukaryotes are composed of exons and introns, their initial transcript precursors undergo AS to remove introns, even some exons in some cases, to generate mature mRNAs [34]. Representative alternative splicing RBPs include serine/arginine-rich (SR) family proteins and hnRNP proteins [35]. Classical SRs bind with RNAs through their RRM domains. Besides, they can also bind to proteins through their serine-rich (RS) enriched C terminal domain [36]. SR proteins facilitates selection of splice site and recruitment of spliceosomal components through recognizing and binding to exonic splicing enhancers [37]. SR proteins can also regulate splicing by interacting with hnRNP proteins [38]. HnRNPs repress splicing by binding to exonic splicing silencers and hindering exon recognition [39].

### mRNA alternative polyadenylation (APA)

APA process adds a poly (A) tail to 3′ untranslated region (UTR) of mRNAs through polyadenylation machinery, a critical process to generate mature mRNA transcripts. Components of the APA machinery are mainly comprised of several RBPs such as cleavage and polyadenylation specificity factor, cleavage stimulation factor, cleavage factor I and cleavage factor II [40]. A number of RBPs have been identified to regulate APA [41]. Except mentioned RBPs, other RBPs such as hnRNPs and SR proteins can also regulate splicing and polyadenylation of mRNAs [35].

### RNA modifications

The process of N^6^-methyladenosine (m^6^A) modification as well as functions of m^6^A are regulated by RBPs. YTH domain-containing protein family members, including YTHDC1–2 and YTHDF1–3, which share the m^6^A-recognizing YTH domain, are the most studied m^6^A readers [42]. YTHDC1, which mainly localizes in the nucleus, regulates RNA transportation and pre-mRNA splicing [43, 44]. YTHDC2, which is ubiquitous in cell cytoplasm, regulates RNA stability and translation [45, 46]. In addition, other proteins such as hnRNPA2/B1 can directly bind to a set of m^6^A bearing pre-mRNAs, elicits mRNA stability and alternative splicing [47].

### RNA stability

RBPs can also regulate RNA stability. Removal of the 5′ cap structure or the 3′ poly (A) tail initiates mRNA degradation process [48]. Up to 8% of mRNA transcripts contain AU-rich elements (AREs) which often reside within 3′ UTR [49]. ARE-related RBPs can recognize AREs and bind with AREs to regulate mRNA stability. For instance, ARE-related RBPs such as AU-binding factor 1 (AUF1), tristetraprolin and human antigen R (HuR) can regulate mRNA degradation or stabilize mRNAs by binding with AREs of corresponding mRNAs [50].

### RNA localization

RNA molecules dwell in specific subcellular locations. Some RBPs affect intercellular localization of target RNAs. Generally, RBPs can recognize and bind to specific sequence in the UTR of target RNAs. After binding, these RBPs recruit and assemble multi-subunit complexes to connect RNAs to cytoskeletal molecular motors, and transport the RNA-protein complexes to their destinations [51]. As a result, dysregulated RBP/RNA complex may disrupt target RNA localization patterns.

### Translational regulation

RBPs play important roles in different stages of translation including translation initiation, elongation, and termination [52]. The binding between RBPs and 5′ UTR or 3′ UTR of mRNAs will result in varying translation efficiency [52]. Besides, an internal ribosome entry site (IRES) can reside in 5′ UTR of mRNAs. RBPs such as IRES transacting factors bind to IRES to trigger translation in a cap structure-independent way [53].

## The involvement of RBPs in treatment resistance of BC

**Fig. 2:**
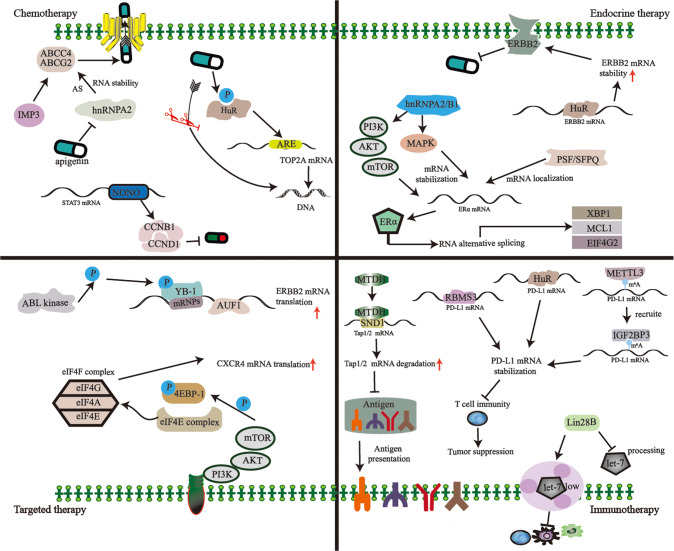
RBPs modulate treatment resistance of breast cancer. The abnormal expression and interaction of RBPs influence treatment resistance by regulating various post-transcriptional events. Representative mechanisms of RBPs regulating resistance of chemotherapy, endocrine therapy, targeted therapy and immunotherapy are shown in respective panel in the schematic diagram.

Systemic therapies for BC are comprised of chemotherapy, endocrine therapy, targeted therapy, radiotherapy, immunotherapy and other approaches. For chemotherapy, the chemotherapeutic agents for treatment of BC include paclitaxel, platinum and anthracyclines in the clinical setting [54]. For endocrine therapy, options include selective estrogen receptor (ER) modulators such as tamoxifen, selective ER down regulators such as fulvestrant, and aromatase inhibitors such as letrozole or anastrozole [55]. For targeted therapy, targeted drugs include anti-HER2 monoclonal antibodies, HER2 antibodies conjunct cytotoxic agents, small molecule tyrosine kinase inhibitors, small molecule targeting CDK4/6 and poly (ADP-Ribose) polymerase inhibitors [56, 57]. Recent studies uncover RBP-mediated regulation of therapy resistance in BC. A table is provided to summarize the alteration of these RBPs in the treatment resistance of BC (Table 2). Schematic diagram of representative RBPs involving in treatment resistance of BC is shown in Fig. 2.

**Table 2:** Altered RBPs in treatment resistance of breast cancer.

RBP	Expression up/down	Targets	Resistant therapy	Functions	Reference
IGF2BP3/IMP3	Up	ABCG2	Doxorubicin	translation	[58]
		PD-L1	Anti-PD-L1	RNA stability	[94]
HuR	Down	TOP2A,	Doxorubicin,	sublocalization,	[61]
	Up	HER2	Tamoxifen	RNA stability	[74]
		PD-L1	Anti-PD-L1	RNA stability	[92, 93]
hnRNPA2	Down	ABCC4, ABCG2	Doxorubicin	AS, RNA stability	[63]
RBMS2	Down	BMF, caspase3, caspase9, PARP	Doxorubicin	translation	[64]
NONO	Up	STAT3, CCNB1, CCND1	Epirubicin,	RNA stability	[66]
PSF	Up	ERα, SCFD2, TRA2B	Tamoxifen	sublocalization	[72]
hnRNPA2/B1	Up	MAPK/AKT signaling	Fulvestrant and tamoxifen	translation	[75]
ERα	Up	XBP1, MCL1, eIF4G2	Tamoxifen	AS, translation	[77]
AUF1	Up	HER2	Trastuzumab	translation	[78]
YB-1	Up	HER2	Trastuzumab	translation	[81, 82]
eIF4E	Up	CXCR4	mTOR inhibitor	translation	[83, 84]
RBM6	Up	Fe65, APBB1	PARP inhibitor	AS	[86]
RBMS3	Up	PD-L1	Anti-PD-L1	RNA stability	[91]
Metadherin	Up	Tap1/2	PD-L1 inhibitor	RNA stability	[97]
Lin28B	Up	let-7 miRNAs	Immunotherapy	AS	[98]

*IMP3* Insulin-like growth factor II mRNA-binding protein 3, *ABCG2* ATP Binding Cassette Subfamily G Member 2, *PD-L1* Programmed cell death 1 ligand 1, *HuR* Hu-Antigen R, *TOP2A* DNA Topoisomerase II Alpha, *HER2* Erb-B2 Receptor Tyrosine Kinase 2, *hnRNPA2* Heterogeneous Nuclear Ribonucleoprotein A2, *ABCC4* ATP Binding Cassette Sub-family C Member 4, *RBMS2* RNA Binding Motif Single Stranded Interacting Protein 2, *BMF* Bcl2 Modifying Factor, *PARP* poly (ADP-Ribose) polymerase, *NONO* Non-POU Domain Containing Octamer Binding, *STAT3* Signal Transducer And Activator Of Transcription 3, *CCNB1* Cyclin B1, *CCND1* Cyclin D1, *PSF* Protein-associated Splicing Factor, *ERα* Estrogen Receptor 1, *SCFD2* Sec1 Family Domain Containing 2, *TRA2B* Transformer 2 Beta Homolog, *hnRNPA2/B1* Heterogeneous Nuclear Ribonucleoprotein A2/B1, *XBP1* X-Box Binding Protein 1, *MCL1* myeloid cell leukemia 1, *eIF4G2* Eukaryotic Translation Initiation Factor 4 Gamma 2, *AUF1* AU-Rich Element RNA Binding Protein 1, *YB-1* Y-box binding protein 1, *CXCR4* CXC chemokine receptor 4, *RBM6* RNA Binding Motif Protein 6, *APBB1* amyloid beta precursor protein binding family B member 1, *RBMS3* RNA binding motif, single-stranded interacting protein 3.

### RBPs regulate chemotherapy resistance of BC

Chemotherapy resistance contributes to BC progression. Anthracyclines are commonly used cytotoxic drugs for BC treatment. Acquired anthracycline resistance is a huge hurdle in BC therapy efficacy. There are several RBPs regulating treatment response of doxorubicin. Insulin-like growth factor II mRNA-binding protein 3 (IGF2BP3/IMP3) depletion substantially reduces the mRNA of ATP binding cassette subfamily G member 2 which functions as an efflux transporter of doxorubicin and then leads to doxorubicin re-sensitivity [58]. HuR has been implicated in the post-transcriptional control of mRNA turnover and stability [59]. HuR has three RRMs and it preferentially interacts with ARE-containing mRNAs [59, 60]. Upon doxorubicin stimulation, HuR is phosphorylated and the phosphorylated HuR regulates doxorubicin target topoisomerase IIa post-transcriptionally to maintain DNA topology and then decreases the efficacy of doxorubicin [61, 62]. HnRNPA2, a RBP proved to regulate AS and RNA stability, plays a critical role in doxorubicin resistance. Repression of hnRNPA2 by a dietary compound apigenin can decrease the expression of efflux transporters ATP binding cassette subfamily C member 4 and ATP binding cassette subfamily G member 2 to induce apoptosis which will promote sensitization of TNBC to doxorubicin [63]. Upregulation of RNA binding motif single stranded interacting protein 2 enhances the sensitivity of BC cells to doxorubicin, while inhibition of this RBP has an opposite trend. Motif single stranded interacting protein 2 positively regulates the expression of Bcl-2 modifier and increases the expression of cleaved caspase 3, cleaved caspase 9 and poly (ADP-ribose) polymerase which in turn contributes to doxorubicin sensitivity [64]. Epirubicin is another anthracycline agent widely used for BC treatment. A novel TNBC-specific RBP, non-POU domain-containing octamer-binding protein (NONO) belongs to the family of Drosophila behavior human splicing [65]. NONO functions in RNA processing, RNA transcription, and DNA repair in cancer [65]. NONO regulates signal transducer and activator transcription 3 post-transcriptionally by binding with its mRNA and the upregulation of NONO causes epirubicin resistance [66]. High expression of eukaryotic translation initiation factor eIF5A2 correlates with decreased doxorubicin sensitivity while silencing of eIF5A2 significantly enhances doxorubicin toxicity [67]. Moreover, there are some RBPs regulating multi-drug resistance. Eukaryotic translation initiation factors have also been shown to play a crucial role in muti-agent resistance. Silencing of eIF4E results in suppression of TNBC growth and sensitization of BC cells to chemotherapeutic drugs of cisplatin, adriamycin, paclitaxel and docetaxel [68].

### RBPs regulate endocrine therapy resistance of BC

Endocrine therapy has been shown to improve outcome of BC patients. However, about 20% of the patients develop endocrine therapy resistance [69]. Abundant evidence shows that RBPs can regulate endocrine therapy resistance [70]. Tumor cells can develop tamoxifen resistance through overexpression or activation of co-activator of ER, suppression of ER or acquiring ER mutation [71]. *ESR1* encodes ER protein. Protein-associated splicing factor closely associates with poor prognosis of ER-positive BC patients and it regulates tamoxifen resistance by up-regulating ERα expression via exporting *ESR1* mRNA from the nucleus to cytoplasm [72]. Knockdown of protein-associated splicing factor also promotes nuclear accumulation of sec1 family domain containing 2, transformer-2 protein homolog beta, suggesting protein-associated splicing factor is a controller of RNA subcellular localization [72]. Similarly, accumulation of HuR in cytoplasm associates with the emergence of tamoxifen resistance. HER2 elevation is one of the characteristics of tamoxifen-resistant ER-positive BC [73]. HuR interacts with 3′ UTR of HER2 transcripts to increase stability of HER2 mRNAs which causes up-regulation of HER2 protein and contributes to tamoxifen resistance [74]. Abnormalities of RBPs also associate with fulvestrant resistance. HnRNPA2/B1 can regulate ERα by stabilizing and increasing splicing efficiency of *ESR1* transcript and trafficking it to the cytoplasm for translation [75]. Under this circumstance, overexpressing hnRNPA2/B1 promotes MCF-7 cell migration and invasion and attenuates endocrine-sensitivity to ER antagonists [75]. According to silico Meta Core network analysis in another research, it is confirmed that transient overexpression of hnRNPA2/B1 promotes processing of pre-miRNAs, which activates TGFβ signaling to induce tamoxifen and fulvestrant resistance [76]. Interestingly, ERα itself is found to be a potent noncanonical RBP which controls RNA metabolism. ERα distributes in cytoplasm and nucleus. In cytoplasm, ERα directly binds with over 1000 mRNAs through a putative RBD located in its hinge region [77]. ERα contributes to endocrine therapies because ERα regulates the AS of X-box binding protein, myeloid cell leukemia 1 and eIF4G2 which are essential for overcoming cellular stress induced by endocrine therapies [77].

### RBPs regulate targeted therapy resistance of BC

AUF1 has been identified to regulate trastuzumab resistance by enhancing HER2 mRNA translation. In acquired trastuzumab-resistant BC cells, trastuzumab treatment will lead to the binding between AUF1 and HER2 mRNA which activates the translation of HER2 and induces trastuzumab resistance [78]. Y-box binding protein 1 (YB-1) participates in mRNA splicing and translation by functioning as a RBP [79]. Phosphorylated YB-1 is an active form of YB-1. When it is not phosphorylated, it binds with messenger ribonucleoprotein particles that inhibit mRNA translation [80]. Once phosphorylated, YB-1 permits the translation of mRNAs of target genes [80]. Phosphorylated YB-1 intensely binds with HER2 mRNA transcript and significantly increases HER2 mRNA translation which will hamper the treatment efficacy of anti-HER2 targeted therapy [81, 82]. Translation repressor eIF4E-binding protein 1 (4E-BP1) can be phosphorylated by mTOR. When phosphorylated by mTOR, 4E-BP1 dissociates from eIF4E complex. After 4E-BP1 is phosphorylated, eIF4F complex conducts translation of specific mRNAs such as CXC chemokine receptor 4 to promote anti-HER2 resistance [83, 84]. RNA-binding motif protein 6 (RBM6), a well-known RBP regulating mRNA AS, is commonly altered in treatment-resistant BC cells [85]. RBM6 controls the AS of Fe65 and amyloid beta precursor protein binding family B member 1 which are essential components of a positive homologous recombination repair regulation complex. Fe65 is drastically decreased when RBM6 is knocked down and the ablation of Fe65 results in impaired homologous recombination of double-strand breaks. Therefore, RBM6-deficient cancer cells are susceptible to poly (ADP-ribose) polymerase inhibition and exhibit sensitivity to cisplatin [86].

### RBPs regulate immunotherapy therapy resistance of BC

Aberrant immune checkpoints helps cancer cells to escape immune attacks [87]. Since recent advances in immune checkpoint blockade inhibitors (CBIs), particularly, CBIs of targeting cytotoxic T lymphocyte associated protein 4 and program death receptor-1 (PD-1) or its ligand PD-L1, immunotherapy appears to be a promising anti-cancer treatment [88]. However, a number of patients did not benefit from CBIs. Metastatic breast cancer patients have a low objective response rate to anti-PD-1/PD-L1 therapy [89, 90]. This could attribute to the abnormal functions of RBPs.

Accumulating evidence shows that RBPs are crucial regulators of PD-L1 and participate in immunotherapy failure. Knockdown of RNA binding motif, single-stranded interacting protein 3 (RBMS3) significantly downregulates the mRNA and protein levels of PD-L1 while overexpression of RBMS3 decreases PD-L1 markedly. RBMS3 stabilizes mRNA of PD-L1 through binding to its AU-rich elements in the 3′ UTR. RBMS3 ablation facilitates anti-tumor T-cell immunity in TNBC [91]. HuR is dysregulated in BC and regulates tumor invasion and metastasis by interacting with a subset of oncogenic mRNAs. Studies show that HuR is also engaged in regulation of PD-L1 post-transcriptionally, as direct binding between HuR and PD-L1 mRNA was observed [92, 93]. In human BC cell lines, HuR knockout expedited PD-L1 mRNA decay and resulted in downregulation of PD-L1 protein level [92]. The protein level of PD-L1 in exosome was also decreased in HuR knockout clones [93]. Additionally, PD-L1 is found to be a downstream target of m^6^A modification mediated by methyltransferase like 3. M^6^A-modified PD-L1 mRNA facilitates the recruitment of IGF2BP3 to improve PD-L1 mRNA stability and promote the expression of PD-L1 [94]. Inhibition of methyltransferase like 3 or IGF2BP3 can enhance the efficacy of immunotherapy through PD-L1-mediated T cell infiltration [94].

In addition to PD-L1, other factors could contribute to the immune-resistance of BC [95]. Metadherin, which is associated with poor prognosis of BC contributes to immunity evasion [96]. Tap1/2 is known as a crucial component of the antigen presentation machinery. Metadherin facilitates the binding between staphylococcal nuclease domain-containing protein 1 and Tap1/2 mRNA and promotes their degradation to hamper anti-tumor immunity [97]. Lin28 is a conserved RBP consisted of two subtypes: Lin28A and Lin28B. Lin28B has been shown to repress let-7 miRNAs by blocking let-7 precursors processing [98]. The role of let-7/Lin28 axis is involved in immunotherapy effectiveness. Upregulation of Lin28B induces BC cells to release low-let-7s-containing exosomes and suppresses the anti-cancer immunity in the pre-metastatic microenvironment [99].

## Therapeutic approaches reversing abnormally expressed RBPs

Since RBPs are critical to cancer treatment resistance, therapeutic approaches reversing abnormal functions of RBPs involved in treatment resistance could restore breast cancer cell sensitivity to anti-cancer treatment. In this part, we show numerous tactics for targeting RBPs in BC and highlight some prospective innovative targeting schemes. (Table 3).

**Table 3:** The potential therapeutic approaches targeting RBPs in breast cancer treatment resistance.

RBP	Therapeutic approaches	Effect	Mechanism	Reference
HuR	azaphilone-9; Dihydrotanshinone-I; KY7123	Induce oxidative stress and DNA damage	Silence HuR expression; interfere with the binding of HuR with mRNA	[100–102]
eIF4E	Ribavirin; 4Ei-1	Inhibit transport and translation of mRNAs such as cyclin D1	Impede eIF4E; antagonize eIF4E cap binding and initiate degradation	[103, 104]
MSI-1	(-)-gossypol; oleic acid siRNA	Reduce Notch/Wnt signaling; elevate p21 expression	Block RBP binding site with RNA; interfere with MSI-1 expression	[106, 107]
RBM38	Pep8	Increase p53 expression	Block the RBM38-eIF4E binding interface	[113]
Lin28	PROTACs	Trigger protein degradation	Link with ubiquitin-proteasome system	[116]

*eIF4E* Eukaryotic Translation Initiation Factor 4E, *MSI-1* Musashi-1, *siRNA* small interfering RNA, *RBM38* RNA Binding Motif Protein 38, *PROTACs* Proteolysis-targeting chimeras.

### Small molecules

Small molecules are often employed to target RBPs. Several small molecular compounds are promising in reversing RBP-conducted treatment resistance. From mentioned above, aberrant expression of HuR is associated with therapy resistance. It regulates chemotherapy and endocrine therapy resistance in BC. Thus, a bunch of small molecular inhibitors targeting HuR have been developed. Compound azaphilone-9 targets HuR primarily by affecting a cluster of RNA-binding residues located near the inter-domain linker region of HuR. Therefore, it can interfere the interaction between HuR and ARE. Since HuR-ARE interactions are essential for stabilizing many mRNAs related to therapy resistance, this disruption could potentially reverse treatment resistance [100]. Dihydrotanshinone-I, derived from the fungal natural product, can disrupt the interaction between ARE and HuR by competing with the binding sites of HuR [101]. KY7123, known as a small molecular HuR inhibitor, can prevent the association between HuR and its target RNAs in vitro and in vivo [102]. EIF4E is involved in muti-treatment resistance as well. Antiviral guanosine analogue ribavirin is found to abrogate eIF4E-mRNA binding by masking the functional site of eIF4E [103]. N-7 Benzyl Guanosine Monophosphate Tryptamine Phosphoramidate Pronucleotide (4Ei-1) is another studied eIF4E inhibitor. 4Ei-1 treatment causes BC cells to re-sensitive to gemcitabine, because 4Ei-1 antagonizes mRNA cap binding ability of eIF4E and initiates eIF4E proteasomal degradation [104]. Musashi RNA binding protein 1 (MSI-1) can enhance treatment resistance by increasing expression of DNA repair-related proteins DNA-PKcs and EGFR [105]. A study employs a fluorescence polarization assay to screen out a small molecule named (-)-gossypol which occupies consensus RNA binding site of MSI-1 and disrupts the binding between MSI-1 and its target mRNAs [106]. Oleic acid binds to the RRM1 of MSI-1 protein and induces a conformational change that inhibits the binding between MSI-1 and its target mRNAs. Oleic acid can also inhibit cell proliferation by upregulating MSI-1 [107].

### siRNAs-based tactics

Small-interfering RNAs (siRNAs) are used to target RBPs to reverse the abnormal expression of RBPs. siRNAs can bind with target genes and subsequently lead to gene silencing. siRNA-based therapies show efficacy and clinical safety during clinical practice. The efficacy of siRNAs has been investigated in several tumor types. After stably transfected with eIF4E-siRNA, cancer cells exhibit decreased levels of VEGF, FGF-2, and cyclinD1 expression. Besides, eIF4E-siRNA significantly inhibits cell growth and promotes cell death by activating caspase 3 in MCF-7 cells. The cytotoxicity of cisplatin is synergistically increased by RNAi-mediated downregulation of eIF4E expression both in vitro and in vivo. This suggests that cisplatin treatment would be more successful when combined with eIF4E-RNAi treatment [108]. Paclitaxel-resistant MDA-MB-231 tumor-bearing mice are re-sensitized to a low dose of paclitaxel by intravenous injections of nanoparticles-loaded eIF4E siRNA [109]. Moreover, HuR siRNA leads to increased reactive oxidative stress and sensitizes TNBC cells to radiation [110]. Increased reactive oxidative stress production is associated with increased DNA damage which contributes to radiosensitivity [110]. Knockdown of HuR by siRNA delays tumor formation and inhibits tumor growth in MDA-MB-231 and SUM159 tumor-bearing mice [111].

### Peptide-based strategy

Peptides enjoy several advantages such as their high specificity, selectivity, tiny size, easy modification and biocompatibility. Different peptides are being designed for cancer treatments and peptide-based strategy is used for reversing therapy resistance [112]. RBM38 can inhibit p53 translation via blocking eIF4E-mediated p53 mRNA translation. Pep8 (8 amino acid peptide) which mimics eIF4E key domain can mask RBM38 binding interface and effectively promote p53 translation, and potently increase p53 expression. Pep8 treatment inhibits tumor spheres growth, colonies formation and xenograft tumors by disrupting RBM38 function [113].

### PROTACs-based strategy

Proteolysis-targeting chimeras (PROTACs), which induce the degradation of proteins via the ubiquitin-proteasome system, are engineered molecules that trigger the degradation of protein of interest [114]. Conventional PROTACs are comprised of three components: a ligand that recognizes protein of interest, a ligand that binds E3 ligase and a linker between them. RBPs are usually undruggable by traditional PROTACs [115]. A new type of PROTACs that can functionally target RBPs is termed RNA-PROTACs [116]. The overexpression of RBP Lin28 has been shown to inhibit the biogenesis of tumor suppressor miRNA let-7 through a direct interaction with pre-let-7 [98, 99]. Based on the established pre-let-7/Lin28 complex, the key partial sequence (AGGAGAU) of pre-let-7 that binds to zinc finger domain of Lin28 is conjugated to a E3-recruiting peptide and this RNA-PROTAC strategy mediates degradation of the Lin28 via the ubiquitination pathway [116]. The establishment of RNA-PROTACs provides a new therapeutic approach to tackle the difficulties of RBP-mediated therapeutic resistance. As RNA-PROTACs are inherently unstable in the presence of nucleases and/or proteases, nanoscale drug delivery systems might be introduced to protect RNA-PROTACs from decay, thereby increasing their biological efficiency in vivo [117].

## Conclusion and perspectives

This review provides an overview of functions of RBPs involved in BC and highlights the important roles of RBPs in the regulation of BC treatment resistance. Both canonical and noncanonical RBPs recognize and interact with their target RNAs by forming RNP complex. RBP inhibitors in combination with other treatments such as chemotherapy, radiotherapy, anti-HER2 therapy and immunotherapy might be a promising therapeutic option for patients who have developed treatment resistance. Therefore, in recent years, RBP-targeted therapies will be attractive to reverse therapeutic resistance. However, on the whole, the complex regulatory network of RBPs is not fully explicit, and much further researches on the role of RBPs in BC are needed.

## Data Availability

All data included in this review are available upon request by contact with the corresponding author.
